# Integrating Molecular Similarity and AlphaFold-Based Structural Alignment for Target Discovery in *Trypanosoma cruzi*

**DOI:** 10.3390/ph19071046

**Published:** 2026-07-07

**Authors:** Albert Ros-Lucas, Nieves Martínez-Peinado, Juan Carlos Gabaldón-Figueira, Maria Morillo-Osorio, Cristina Ballart, Montserrat Gállego, María-Jesús Pinazo, Joaquim Gascón, Ana Requena-Méndez, Julio Alonso-Padilla

**Affiliations:** 1 ISGlobal, 08036 Barcelona, Spain; 2 Campus Mar, Universitat Pompeu Fabra (UPF), 08003 Barcelona, Spain; 3 CIBERINFEC, ISCIII–CIBER de Enfermedades Infecciosas, Instituto de Salud Carlos III, 28029 Madrid, Spain; 4 Secció de Parasitologia, Departament de Biologia, Sanitat i Medi Ambient, Facultat de Farmàcia i Ciències de l’Alimentació, Universitat de Barcelona, 08028 Barcelona, Spain; 5 Department of Clinical Microbiology, Hospital de la Santa Creu i Sant Pau, 08025 Barcelona, Spain; 6 Drugs for Neglected Diseases Initiative (DNDi), Rio de Janeiro 20010-020, Brazil; 7 Department of Medicine Solna, Karolinska Institute, 171 77 Stockholm, Sweden; 8 INTERTRYP, University of Montpellier, CIRAD, IRD, 34398 Montpellier, France

**Keywords:** *Trypanosoma cruzi*, Chagas disease, target identification, molecular similarity, AlphaFold, structural alignment, neglected tropical diseases, drug discovery

## Abstract

**Background**: Chagas disease, caused by the parasite *Trypanosoma cruzi*, remains a major neglected tropical disease, with millions of people living with the infection worldwide. Current treatments are effective in the acute stage of the disease, but are poorly tolerated and show reduced efficacy in chronic infections, highlighting an urgent need for novel therapeutic strategies. A key bottleneck in early-stage drug discovery is target identification, which is traditionally dependent on costly and low-throughput experimental methods. Computational approaches offer a cost-effective and fast alternative to traditional methods. **Methods**: In this study, we present an integrated in silico pipeline that combines ligand-based and structure-based computational approaches to prioritize potential molecular targets for bioactive compounds against *T. cruzi*. The ligand-based component performed similarity searches across curated bioactivity databases containing known ligand–protein associations, and the most similar candidates were then further evaluated using a structure-based approach through pairwise structural alignment against the *T. cruzi* proteome from AlphaFold. **Results**: The pipeline was validated using eight compounds with known targets, successfully recovering the correct target in six cases. Additionally, two compounds with anti-*T. cruzi* activity but unknown mechanisms of action were analyzed to hypothesize their potential targets. **Conclusions**: Overall, the pipeline demonstrated moderate success, with limitations arising from challenges in handling novel chemotypes and poorly annotated targets. Nevertheless, its modular nature allows for an easy adaptation to other neglected tropical diseases, providing a flexible and cost-effective framework for early-stage target prioritization.

## 1. Introduction

Chagas disease (CD) is a potentially life-threatening neglected tropical disease (NTD) caused by the protozoan parasite *Trypanosoma cruzi*. The disease remains endemic in 21 countries across Latin America, which carry the greatest burden in terms of morbidity and mortality. Moreover, CD has expanded beyond Latin America and is now emerging in North America, Europe, and parts of the Western Pacific due to migration flows. Current estimates indicate that approximately 10.5 million people live with the infection worldwide, with around 8000 annual deaths [1,2,3]. To date, no vaccine against CD has reached approval for clinical use, despite ongoing research efforts [4]. Antiparasitic treatment relies on two drugs developed in the 1970s: nifurtimox (NFX) and benznidazole (BNZ). These drugs are most effective when administered during the acute phase and for congenital infection, when this is diagnosed in time. However, their efficacy in chronic infections is limited, and both require prolonged administration regimes that frequently associate with adverse effects, which can negatively impact treatment adherence [5]. Consequently, the development of safer and more effective therapeutic options remains an urgent priority.

Traditional drug discovery and development is a lengthy, costly, and high-risk process. On average, it can take over a decade and cost upwards of 1–2 billion USD, with attrition rates exceeding 90% [6]. Early identification of molecular targets is critical to improving success rates [7]. This is crucial in drug development for NTDs, where limited funding makes late-stage failures very costly. A striking illustration within the CD clinical research field is the failure of CYP51 inhibitors. The triazoles posaconazole and ravuconazole (as its prodrug E1224), which target the *T. cruzi* sterol 14α-demethylase (CYP51) enzyme, demonstrated moderate to potent efficacy in early developmental stages [8], but both proved inferior to BNZ in clinical trials [9,10]. This disconnect between preclinical efficacy and clinical performance led to CYP51 inhibitors being actively deprioritized in current drug development pipelines against *T. cruzi* [11].

Historically, target identification has relied on experimental techniques such as expression cloning, protein microarrays, RNA interference (RNAi), CRISPR-based screening, and ligand-binding assays [12]. These approaches are often expensive and limited in throughput, making target identification a bottleneck for the development of new drugs for NTDs such as CD, where research funding is scarce and commercial incentives remain low [13]. In recent years, computational approaches have become essential tools in drug discovery [6]. Among the many stages for which these methods look promising, target identification would be a particularly impactful application. Computational strategies for target deconvolution can be broadly classified into ligand-based and structure-based. Although they rely on different underlying assumptions, these two strategies are often complementary and can be effectively combined to strengthen prediction accuracy. Ligand-based approaches rely on information from known active compounds to infer potential targets, using techniques such as similarity searching, pharmacophore modeling, and quantitative structure–activity relationship (QSAR) analysis [14]. While powerful, these methods are dependent on the availability of previously characterized ligands. On the other hand, structure-based approaches exploit the three-dimensional architecture of proteins to predict interactions with small molecules. For example, molecular docking enables high-throughput screening of ligand–protein interactions, and can be used for target identification using inverse virtual screening [15]. However, access to accurate 3D protein structures is a key requirement, and those are traditionally obtained experimentally. For organisms such as *T. cruzi*, the availability of biological structures has historically been very limited, restricting the application of molecular docking to a handful of targets. This limitation has been largely overcome by recent advances in artificial intelligence, particularly the development of AlphaFold [16]. AlphaFold can accurately predict 3D structures directly from a protein primary sequence, achieving near-experimental accuracy in many cases. Currently, the AlphaFold Protein Structure Database [17] includes tens of thousands of predicted models for *T. cruzi* and other key organisms of global health interest, facilitating large-scale analyses that were previously not feasible.

In this study, we developed an in silico pipeline that integrated ligand- and structure-based approaches to leverage the strengths of both strategies. The ligand-based component queried a custom database built from open repositories such as ChEMBL, BindingDB, and Protein Data Bank (PDB) to identify structurally similar compounds to a query molecule. These candidates were then subjected to structure-based analysis through pairwise structural alignment against *T. cruzi* proteome models obtained from AlphaFold. The aim of this study was to prioritize and narrow the list of potential molecular targets for a known bioactive compound, allowing experimental efforts to be more focused and cost-effective.

## 2. Results and Discussion

Target identification is a critical step in early drug discovery, as it establishes the biological foundation upon which all subsequent development depends. Choosing an appropriate molecular target directly influences the chance of success, as poorly validated targets are a major contributor to late-stage clinical failure [18,19]. Several approaches have been used to determine molecular targets for bioactive compounds. High precision experimental methods such as RNAi and CRISPR-based screenings can be used to link gene disruptions with phenotypic outcomes, linking possible resistance and/or susceptibility with a certain compound, thus defining a molecular target. However, these are resource-intensive methods, and especially in the case of *T. cruzi*, limited by the parasite’s biology [20,21]. Biochemically based methods can confidently identify interactions between compounds and proteins, but typically require prior knowledge of a compound’s mechanism of action, and thus are only useful when working with analogs of known-target compounds. Computational approaches such as ligand-based similarity searches, QSAR models, and structure-based techniques such as inverse virtual screening, can provide a fast and cost-effective way of identifying the compounds’ likely targets [22,23]. The main drawback is that they depend on the availability and quality of existing data (ligand–protein affinity profiles, protein structures, etc.), and require subsequent experimental validation. The present study demonstrates the potential of exploiting the strengths of both ligand-based and structure-based computational approaches into a unified pipeline for molecular target identification in the protozoan *T. cruzi*. To date, no similar tools have been developed specifically for this parasite. In fact, most existing computational target prediction platforms (e.g., SwissTargetPrediction, ReverseDock, PPB2) mainly focus on human and other model organisms’ proteomes, leaving neglected pathogens such as *T. cruzi* largely uncovered [24,25,26].

Firstly, a curated database drawing on ChEMBL [27], BindingDB [28], and PDB with PDBBind [29,30] was constructed to provide a wealth of ligand–protein affinities and interactions. The protein–ligand database was composed of 877,824 unique compounds after filtering and deduplication strategies were applied, with associated data for 30,969 unique protein entries (Table 1). Importantly, this ligand database was not restricted to compounds previously tested against *T. cruzi* targets, and it was enriched with knowledge from better studied organisms such as humans, mice or rats. A hit search based on similarity was then carried out by combining two complementary molecular fingerprints. Using molecular similarity as a tool for target deconvolution is grounded in the well-established principle that compounds with similar structures and physicochemical properties tend to share biological targets [31]. Pattern fingerprints, which are unique to RDKit [32], are designed to be used in substructure screening. They identify structural features by matching a set of generic SMARTS patterns, and then hashing each match according to local atom and bond types. This fingerprint type is used in both whole-molecule and Bemis–Murcko scaffold comparisons. ErG fingerprints, by contrast, abstract the molecule into pharmacophoric features (hydrogen donors and acceptors, ionizable atoms, etc.) and encode the distances between them [33]. The weighted score used (see Section 3.3) reflects a deliberate design choice: Pattern fingerprints, which dominate the score, reward direct structural resemblance, while ErG fingerprints ensure that compounds sharing pharmacophoric features are not penalized for structural differences. Using this approach, the ligand database could be ranked by similarity to a compound of interest. In this pipeline, a threshold was established retaining the top 20 compounds by similarity for downstream analysis, a very stringent cutoff equivalent to the top 0.065% of compounds (20/30,969, given that only the most similar target was kept per ligand). This strict cutoff was aimed at minimizing false positives and reducing the manual curation further down the process.

In a second stage, protein targets associated with the hits identified through the similarity search (a given compound might have more than one target associated with it) were used for structural alignments against predicted *T. cruzi* protein models from the AlphaFold Protein Structure Database. The current availability of structures for previously unsolved *T. cruzi* proteins facilitates a new approach to CD drug discovery. Before AlphaFold, structure-based analyses were limited to the relatively small subset of *T. cruzi* proteins for which experimental structures had been solved. Currently, on PDB there are 463 entries for *T. cruzi*, which correspond to 273 distinct proteins (by UniProt accession). By contrast, using a wide collection of predicted *T. cruzi* structures allows for a more exhaustive and less biased target search, albeit with the downside of working with computational predicted models rather than with experimentally validated structures. From the approximately 19,000 *T. cruzi* protein models available in AlphaFold, a curated subset was selected using a filtering pipeline designed to balance structural quality, targetability, and computational efficiency (Supplementary Figure S1).

To ensure functional relevance, structures lacking any InterPro domain annotations were removed, which excluded approximately 7000 proteins, and highlights the systematic annotation gaps in the parasite’s proteome. Second, sequence clustering was used to reduce redundancy, to avoid over-representation of multi-gene families and to account for the diploid nature of the hybrid CL Brener strain [34], the proteome available in the AlphaFold database. Following this, pocket prediction on the remaining 7089 proteins identified 311,498 putative binding sites. Given the large number of false binding sites that were probably predicted, only those with a P2Rank score above the mean were kept. Stringent structural quality filters were applied to the remaining AlphaFold models at a local level, to ensure that binding sites were structurally sound. This avoided discarding intrinsically disordered proteins in cases they contained ordered domains with predicted binding sites. Finally, only pockets with at least 50% of its residues belonging to an InterPro domain were kept. This resulted in 5570 high-quality *T. cruzi* models with 28,919 identified putative binding sites, with a median of four pockets per protein. By performing both global and local (using the predicted binding sites) structural alignments, the pipeline identified parasite proteins that share not only overall structural similarity with potential targets, but also local pocket structure similarity. In addition, relying on a structure-based alignment algorithm such as TM-align [35] ensured that comparisons were effectively sequence-independent, which is very useful given the evolutionary distance between *T. cruzi* and most studied model organisms [36].

**Figure 1:**
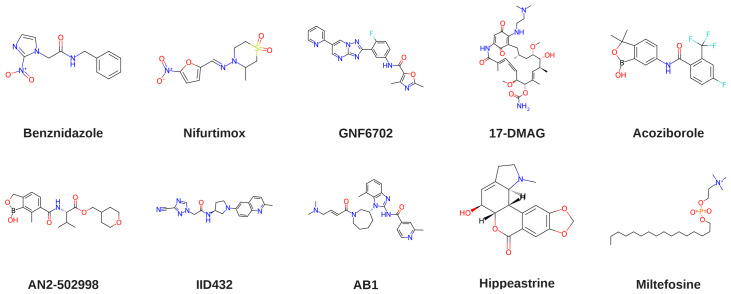
Compounds used in the pipeline.

A collection of compounds was selected to assess the performance of the pipeline. This set included a selection of agents with known anti-*T. cruzi* activity with both known and unknown molecular targets (Figure 1, Table 2). Both standard antiparasitic CD treatments (NFX and BNZ), and compounds under development such as GNF6702, 17-DMAG, acoziborole, AN2-502998, IID432, and AB1, were used as ‘controls’ due to their molecular targets being largely described. Hippeastrine and miltefosine, which lack properly identified targets, served as interesting cases for which molecular targets could be elucidated.

For each of the compounds selected to test the pipeline (Figure 1, Table 2), a similarity search was carried out to find the top 20 most similar ligands from the database. The associated protein or proteins were then screened against the *T. cruzi* proteome by structural alignment. Some ligand–protein pairs gave neither global nor local alignments to any *T. cruzi* protein with a TM-align score of at least 0.5, the lower cutoff used for defining a shared topology [46], and thus were not reported. For each ligand–protein pair found, results were filtered so that only the best predicted pocket (by score) of the protein was considered (not applicable for PDB entries), *T. cruzi* results were then sorted by global and local structural alignment scores, followed by predicted pocket scores. A positive recovery was considered as such when at least one protein in the resulting shortlist corresponded to the described *T. cruzi* target or shared a high degree of similarity. Raw results can be found in Supplementary File S1, while the most meaningful results are summarized in Table 3 and discussed in detail.

**Figure 2:**
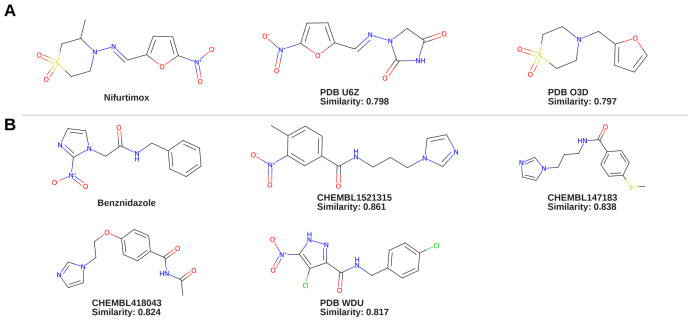
Most relevant chemical hits for nifurtimox (**A**) and benznidazole (**B**).

Analysis of the results for the control compounds indicates that the pipeline was moderately successful. NFX and BNZ, the only drugs approved for the treatment of CD at present, have been classically described as pro-drugs targeting the parasite nitroreductase (NTR) enzyme, their NTR-metabolized forms acting as oxidizing agents that ultimately damage parasite cells [47]. In the case of NFX (Figure 2A), the Q4DCW9 NTR enzyme was identified among the top matches through the compound hit from PDB U6Z, linked to the PDB entry 7NB9 that corresponds to the antibiotic nitrofurantoin bound to the *E. coli* NfsA (oxygen-insensitive nitroreductase). Nitrofurantoin is structurally similar to NFX, sharing the nitrofuran moiety, but has a hydantoin substituent in place of NFX’s thiomorpholine-like ring. Structural alignment revealed that the Q4DCW9 NTR shares only 18% global sequence similarity with the *E. coli* NfsA. A second target of interest, Q4CT43 trypanothione reductase, was identified via PDB ligand entry O3D, linked to PDB entry 8PL9 of a thioredoxin glutathione reductase from *Schistosoma mansoni*. Although the O3D ligand lacks the nitrofuran ring of NFX and consists only of a furan and thiomorpholine-like ring, trypanothione reductase has independently been proposed as a NFX target [48].

By comparison, no NTR was recovered for BNZ, likely because few such enzymes populated the database beyond the *E. coli* NfsA described above. In total, only 18 ligands were associated with NTR entries, corresponding to just 11 distinct proteins. Nevertheless, the pipeline identified other targets previously associated with BNZ (Figure 2B). For instance, *T. cruzi* aldo/keto reductase has been described as a BNZ target [49,50], and the 20th-ranked hit, PDB ligand WDU from entry 4WDU, corresponding to human type 5 17β-hydroxysteroid dehydrogenase (17β-HSD5), led via structural alignment to the *T. cruzi* Q4D831 aldo/keto reductase. Although this hit sits at the boundary of the top-20 cutoff, the threshold applied was very strict, given the database size, and the global TM-align score of 0.93 confirms that Q4D831 is a strong structural homologue of 17β-HSD5. Furthermore, cytochrome P450 enzymes have also been proposed as possible targets and mechanisms of BNZ resistance [51], and the Q4D0S6 cytochrome P450 appeared after the alignments in three independent queries from three distinct chemical hits (CHEMBL1521315 with human aromatase P11511, CHEMBL147183 with *R. norvegicus* thromboxane-A synthase P49430, and CHEMBL418043 with human thromboxane-A synthase P24557).

**Figure 3:**
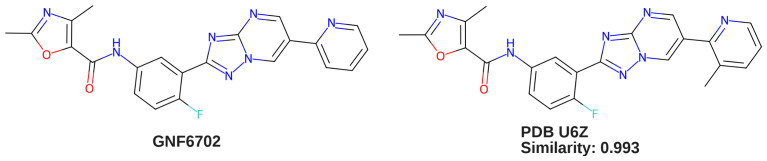
GNF6702 and PDB U6Z (LXE408).

GNF6702 was identified through Novartis high-throughput screening campaigns against kinetoplastid parasites [52], belonging to a new class of anti-trypanosomatid molecules that inhibit the proteasome with high efficacy against *T. cruzi*, *T. brucei*, and *Leishmania donovani* [38]. An almost identical match was found in PDB ligand N2E (Figure 3) from entry 6TCZ, corresponding to LXE408 complexed with the *L. tarentolae* 20S proteasome subunit. Indeed, LXE408 is an optimized analog of GNF6702 with improved ADME properties, differing only by a methyl group at the pyridine 3-position [53]. Two *T. cruzi* targets were identified through alignment: proteasome subunit beta type-2 (from chain K of 6TCZ) and proteasome subunit beta type-5 (from chain L), both with TM-align scores close to 1 in global and local alignments.

**Figure 4:**
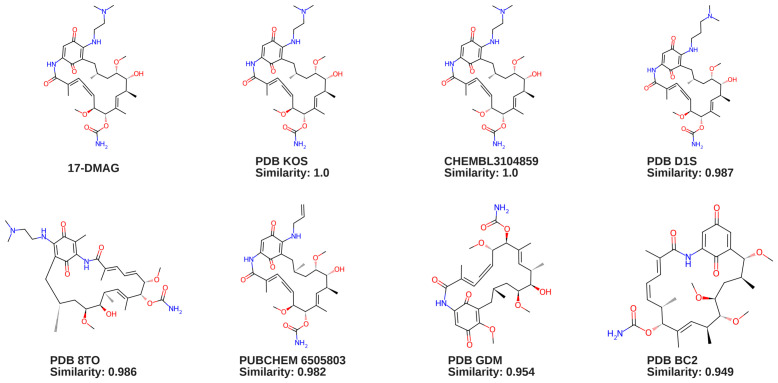
Most relevant chemical hits for 17-DMAG.

17-DMAG is a geldanamycin derivative with antitumor properties that targets the chaperone heat shock protein 90 (Hsp90). Several chemical hits to 17-DMAG were identified (Figure 4), including its direct structural match (CHEMBL3104859 and PDB KOS), closely related compounds such as 17-DMAP-geldanamycin (PDB D1S), and other members of the ansamycin family (PDB 8TO, CHEMBL109480, PUBCHEM 6505803, PDB GDM, PDB BC2). In all cases, structural alignments recovered heat shock proteins from *T. cruzi*, predominantly Hsp90 (Q4DW89) and Hsp85 (Q4DBM7), with one additional match (Q4E2Q2) arising from alignment with human mitochondrial Hsp75. Notably, 17-DMAG has also been reported to inhibit JmjC histone lysine demethylases (KDMs) [54]. Two alignments with human lysine-specific demethylases 5A and 6B recovered the *T. cruzi* JmjC domain hydroxylase Q4CXU5, but only through local alignments (Supplementary File S1). This suggests that while the overall protein fold diverges considerably from human KDMs, the binding site architecture is conserved, suggesting an alternative target of 17-DMAG in the parasite. This example illustrates the complementary value of local structural alignments, which can identify biologically relevant associations that may be missed by global comparisons when overall similarity is low. Given their low computational cost, their inclusion in the pipeline provides a cost-effective boost in discriminatory power.

**Figure 5:**
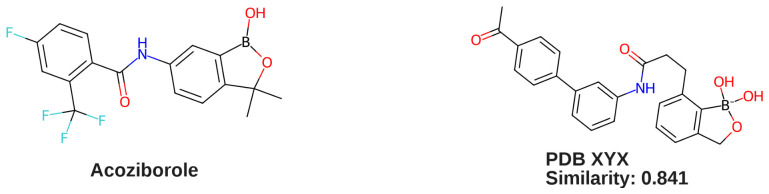
Acoziborole and PDB XYX.

Acoziborole and AN2-502998 are benzoxaboroles that target cleavage and polyadenylation specificity factor subunit 3 (CPSF3), an endonuclease involved in pre-mRNA processing. A CPSF was found by the pipeline for acoziborole with PDB ligand XYX (Figure 5) ranked in the top 10, corresponding to PDB entry 8T1Q of the human CPSF73 catalytic segment, from which structural alignment identified the *T. cruzi* Q4DR37 CPSF. However, no CPSF protein was returned for AN2-502998, likely reflecting limited binding affinity data. Only three PDB ligands are found together with human CPSF3: JBG, XYX and XZC, the latter with the highest similarity to AN2-502998, but sitting at position 39. In addition, a manual search in ChEMBL shows only two compounds described to bind to CPSF, with structures very different from those of the benzoxaboroles. All this suggests that CPSF is an understudied target, which translates into few entries in the database, possibly explaining the lack of matches for AN2-502998.

Compound IID432 is a recently described cyanotriazole that effectively inhibits the *T. cruzi* topoisomerase II [42], but the pipeline was unable to recover this target. The compound from which IID432 is derived, called CT1 [55], is found as PDB YWX together with entry 8GCC of the *T. cruzi* topoisomerase II. However, although these compounds are related and share the cyanotriazole moiety, they differ in the rest of the molecule, and CT1 is found in position 26, just out of the top-20 cutoff established. A more lax threshold could potentially be used, but at the cost of introducing false targets into the shortlist of results.

**Figure 6:**
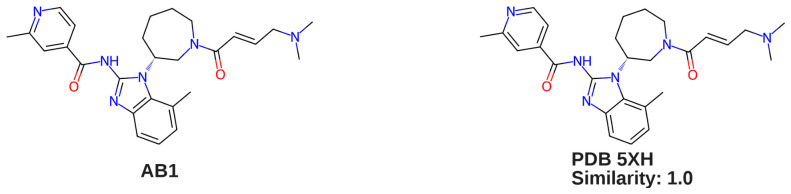
AB1 and PDB 5XH.

AB1 is an amidobenzimidazole protein kinase inhibitor, described as targeting the trypanosome kinetochore, specifically the CLK1 protein also known as kinetoplastid kinetochore protein 10 (KKT10). This compound was itself found in the constructed database as a hit (PDB 5XH, Figure 6) and related to two PDB entries, 5FEQ together with an EGFR kinase domain, with the highest ranking alignment being the Q4D5A5 serine/threonine-protein kinase NEK21, and 6Q2A together with the *T. brucei* CLK1 kinase domain, with the highest match returning the Q4E3Z0 kinetoplastid kinetochore protein 19. KKT19 is also described as CLK2, which is highly related to CLK1, sharing an identical protein kinase domain, and AB1 is reported to probably inhibit its function too [43]. The prioritization of KKT19 over KKT10 in the structural alignment might well be caused by small differences in disordered regions in the AlphaFold models, as upon inspection both have a high TM-align score (0.9855 and 0.9846, respectively).

**Figure 7:**
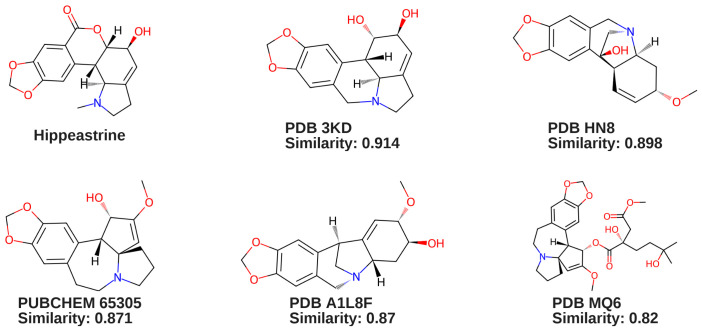
Most relevant chemical hits for hippeastrine.

Two compounds with unknown targets were introduced in the pipeline, hippeastrine and miltefosine. Hippeastrine is an alkaloid synthesized by plants of the Amaryllidaceae family, which was described as having anti-*T. cruzi* activity [44]. Interestingly, four of the chemical hits with the highest identity (PDB 3KD, HN8, A1L8F and MQ6, corresponding to the similar alkaloids lycorine, haemanthamine, montanine and harringtonine) related to PDB entries complexed with eukaryote 80S ribosomes (Figure 7). Given the relatively high selectivity index of hippeastrine in Vero and HepG2 cells [44], it might be that it specifically targets parasite ribosomes. Indeed, trypanosomatid ribosomes have been considered a potential drug target due to some unique features in their structure [56,57]. Even though our pipeline is designed to solely identify protein targets through structural alignment, it can also detect cases where a ligand is bound predominantly to nucleic acids in a PDB structure. This is assessed by analysing the local environment of the ligand when establishing the binding site: if ≥75% of the nearby molecules are DNA or RNA, the interaction is classified as binding to nucleic acids rather than proteins. In these cases, the result is still reported, but annotated as ‘interacting with nucleic acids’ instead of a protein target. Another interesting hit was PubChem CID 65305, corresponding to the alkaloid homoharringtonine (also known as cephalotaxine), which is described as a potent inhibitor of the human Q16539 mitogen-activated protein kinase 14 (MAPK14), also known as p38α [58]. The resulting alignments highlighted the *T. cruzi* Q4D3A0 mitogen-activated protein kinase 2 as a putative target of hippeastrine, a potential drug target for the parasite previously described for its role in regulating differentiation and proliferation [59].

**Figure 8:**
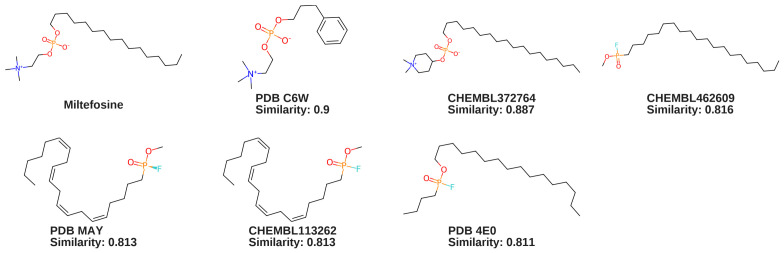
Most relevant chemical hits for miltefosine.

Miltefosine is a synthetic alkylphosphocholine characterized by a long hydrophobic hydrocarbon chain and a polar head. The hydrocarbon chain appears to be poorly captured by the combination of fingerprints used, resulting in some hits being macrocycles or just sharing the polar head (Supplementary File S1). Nevertheless, some interesting hits were retrieved (Figure 8). The top-ranked chemical hit, PDB ligand C6W (cyclofos-3, a detergent that shares only the phosphocholine polar head group with miltefosine), is co-crystallized with a *S. pneumoniae* thioredoxin (PDB 2YP6), and the subsequent structural alignment identifies the *T. cruzi* Q4CX87 peroxiredoxin as a potential homologue. Peroxidoxins are important antioxidant proteins, and their hypothetical perturbation by miltefosine would be consistent with the oxidative stress the drug is known to induce [60]. A second hit, CHEMBL372764, is reported to act on the human P15374 ubiquitin carboxyl-terminal hydrolase isozyme L3, for which the best *T. cruzi* alignment corresponded to the Q4D1R0 cysteine peptidase, Clan CA, family C12 (ubiquitin carboxyl-terminal hydrolase). These proteins belong to the broad family of cysteine peptidases, which have been extensively studied in *T. cruzi* as potential targets, such as the case of the widely studied cruzipain [61]. Indeed, studies performed in *L. donovani* promastigotes report that proteases are involved in the apoptosis-like death induced by miltefosine [62], thus this mode of action warrants further study in *T. cruzi*. Another group of potential targets involve lipid metabolism, with three chemical hits, CHEMBL462609, CHEMBL113262, and PDB ligand 4E0, all resulting in the *T. cruzi* Q4CSM0 monoglyceride lipase, which splits monoacylglycerides into glycerol and free fatty acids, and hits CHEMBL462609, CHEMBL113262, and PDB ligand MAY, which from mouse, human and rat fatty-acid amide hydrolases, respectively, identify the Q4D5K8 amidase as a putative target. Given that one of the described mechanisms of action of miltefosine in *T. cruzi* involves disruption of lipid metabolism [47], the inhibition of these enzymes would be mechanistically consistent.

Despite its success, our pipeline has several limitations that must be acknowledged. Since the ligand database is built on previous public chemical databases, compounds with cutting-edge scaffolds or unusual chemotypes are unlikely to find informative hits. Also, even small chemical modifications can substantially modify target specificity, which means that chemical similarity between a query compound and a database match does not guarantee shared biology. The weighted similarity score relies on a heuristic weighting scheme that was defined a priori rather than systematically optimized. While the chosen weights reflect the intended balance between structural and pharmacophoric similarity, they may not represent the most optimal combination. Future work could investigate different weighting approaches or fingerprint combinations using larger and independent benchmark datasets. Likewise, the top-20 cutoff was chosen heuristically to balance sensitivity and the practical burden of manually assessing false positives, while maintaining reasonable computational efficiency as each additional hit introduces one or more candidate targets requiring structural alignment. Although this threshold performed satisfactorily in the present study, its selection was not systematically optimized, and future work should evaluate recovery rates across a range of cutoff values to determine the optimal cutoff.

For targets, there is an inherent bias toward well-studied protein families, such as kinases, GPCRs and proteases, which are disproportionately represented in databases such as ChEMBL and consequently appear frequently in the pipeline results, inflating their matches (Supplementary Figure S2). Conversely, novel or understudied targets such as CPSF3 are at a disadvantage in the similarity search, given their lack of ligand–target records in public databases. This reflects a general limitation of database-based approaches, which are inherently constrained by the quality and completeness of the data sources. Notably, this could be somewhat mitigated by the local alignments strategy, which focuses on binding sites, and can sometimes find potential matches if binding site topology is shared. Nonetheless, on their own they cannot guarantee a shared biological function, and such results should be interpreted within the biological context. Another limitation is the quality of AlphaFold models, as it varies considerably across the *T. cruzi* and other organisms’ proteomes, for example, with intrinsically disordered proteins and multi-domain complexes. This can affect both the quality of structural alignments and the reliability of binding site predictions. Finally, as illustrated by the case of hippeastrine, the pipeline is designed to identify protein targets, which hinders its capability to find nucleic acid-rich targets such as ribosomes and other ribonucleoproteins. However, for ligands originating from PDB, it can still recognise when binding occurs predominantly to nucleic acids by analysing the ligand’s local environment. The interaction is then classified and reported as associated to nucleic acid rather than to a specific protein target.

## 3. Materials and Methods

### 3.1. Ligand–Target Pairs Database Construction

We constructed a comprehensive ligand–protein binding database by combining data from three sources: ChEMBL (version 36) [27], BindingDB [28], and the PDB [29] together with PDBBind (2020 release) [30]. From the ChEMBL database, we extracted binding affinity measurements using stringent filters. We restricted our analysis to binding assays targeting either single proteins or protein complexes, selecting only those with the highest possible score (9 and 7, respectively). For protein complexes, each subunit was treated as an individual ligand–protein pair. Only measurements with exact values in nanomolar units were retained (i.e., excluding measurements with ‘>’ or ‘<’). We included four standard activity types: $K_{i}$ (inhibition constant), $K_{d}$ (dissociation constant), IC_50_ (half-maximal inhibitory concentration), and EC_50_(half-maximal effective concentration). Records marked as potential duplicates or containing validity flags suggesting unreliable measurements were excluded. Additionally, records with activity comments indicating undetermined, inconclusive, or inactive results were removed. Only entries with available pChEMBL values and that were either manually validated or without validity comments were retained, ensuring high-quality binding data. Protein targets were kept with their UniProt accession IDs [63]. For BindingDB, we extracted binding affinity data for $K_{i}$, $K_{d}$, IC_50_, and EC_50_ measurements, also keeping only those with exact measurements, and retaining the primary UniProt accession number for each target. Finally, for the PDB data, we combined ligand data from the Chemical Component Dictionary, downloading the OpenEye stereo SMILES, with the PDB relations of each ligand to its PDB entry with a script provided by the RCSB [64]. Additionally, binding data from PDBBind was incorporated for existing entries, with the UniProt accession number provided by the SIFTS PDB chain-to-UniProt mapping [65]. All entries underwent molecular standardization using RDKit (version 2025.09.6) [32]. The SMILES of each compound were validated to discard invalid strings. Molecules with more than one fragment (e.g., salts) were split and only the largest fragment was considered, keeping it if it had more than 10 and fewer than 50 heavy atoms. Additionally, only compounds composed exclusively of common organic atoms were retained (H, B, C, N, O, F, P, S, Cl, Se, Br, I). Finally, canonical SMILES representations as well as InChI keys were generated.

The integration of multiple databases required a deduplication strategy to handle redundant measurements. First, within each source database, we deduplicated compound–target pairs by selecting the measurement with the highest priority ($K_{i}$/$K_{d}$ > IC_50_/EC_50_). For ties in measurement type, we selected the entry with the lowest binding affinity value. Only pairs with a $K_{i}$, $K_{d}$, IC_50_, or EC_50_ below 1 µM were considered as ’active’ against their target protein, and kept. In the case of PDB data, entries without PDBBind affinity values were kept regardless. Second, after combining all three databases, we performed a final deduplication step to select a single measurement per unique compound–target pair. For compound–target pairs appearing in multiple databases, we prioritized PDB/PDBBind over ChEMBL and BindingDB, given the relevance of protein structures in our pipeline, and using the same measurement type and affinity value criteria described above in case of a tie. This approach ensured that each compound–target interaction was represented by a single, high-quality measurement in the final integrated database. In order to carry out similarity-based searches, SMARTS patterns (Pattern) (2048 bits) [32] and Extended-Reduced Graph (ErG) [33] fingerprints were generated for each compound using RDKit. In addition, Bemis–Murcko scaffolds were extracted using RDKit’s implementation of Murcko decomposition, and Pattern fingerprints were calculated.

### 3.2. Trypanosoma cruzi Protein Structure Selection

The whole *T. cruzi* proteome available on the AlphaFold Protein Structure Database was downloaded in February 2026 (v6). UniProt IDs were obtained from each structure file and used to retrieve annotations from the InterPro database [66]. Domain annotations were filtered to retain only entries corresponding to three annotation types: ‘domain’, ‘family’, and ‘homologous superfamily’. For each retained domain, we extracted the InterPro accession ID, domain type, domain name, and sequence coordinates (start and end positions). In cases with multiple instances of the same domain in a protein, the lowest and highest positions were kept as start and end, respectively. Only proteins with InterPro annotations were kept for the rest of the pipeline.

Sequences obtained from the AlphaFold PDB files for proteins with InterPro annotations were clustered using CD-HIT [67] at 90% identity threshold in order to reduce redundancy. Only one protein (the reference) per cluster was kept. The structures of each of these proteins was used in fpocket 4.0 [68] to predict binding sites, which later were re-scored using P2Rank 2.4 [69]. Pocket centroids were calculated as the arithmetic mean of all Voronoi vertex coordinates obtained from fpocket. To further characterize potential binding pockets, AlphaFold-derived predicted local distance difference test (pLDDT) and predicted aligned error (PAE) measurements were obtained from each structure. For each binding site, both mean and median pLDDT values were calculated across all pocket-lining residues, as well as the mean PAE between all pairs of residues, with an overall pocket PAE computed as the mean across all pocket residues. Finally, the proportion of residues that pertained to any given InterPro domain was calculated, keeping the maximum domain proportion across all domains of the protein. Annotation of these proteins was obtained from TriTrypDB [70].

### 3.3. Similarity Search, Hit Selection and Structural Alignments

For a given query compound, molecular fingerprints and scaffolds were generated using the same methods as in the database. The similarity of the query compound to the rest of the database compounds was calculated using the cosine similarity metric, calculating a weighted similarity score as follows:

(1) similarity_score=0.5×pattern_similarity+0.25×ErG_similarity+0.25×scaffold_pattern_similarity

These weights were selected heuristically based on the complementary information captured by the descriptors rather than through optimization on the validation set, in order to avoid introducing overfitting or biases. Greater weight was assigned to Pattern fingerprints because they primarily capture structural similarity, while ErG fingerprints were included to account for pharmacophoric similarity between structurally diverse compounds. Hit compounds were then ranked by their composite similarity score, and the top 20 hits were considered for further analysis. If a hit compound had more than one target described, all those that passed the affinity threshold were considered.

To find putative *T. cruzi* targets, pairwise protein structural alignment was carried out between each identified target from a hit, using either their PDB (if available) or AlphaFold structures, and the selection of *T. cruzi* AlphaFold structures. Reported targets corresponding to viral proteins without available PDB entries were excluded from the analysis, as AlphaFold structures were not available. Alignments were performed using the TM-align algorithm (within the US-align suite) [35]. Only *T. cruzi* proteins with predicted binding sites were used. To filter out low quality binding sites, the P2Rank scores of all pockets were evaluated, and those scoring below the overall dataset mean (4.8) were excluded from further analysis. In addition, pockets were filtered to have at least a mean pLDDT score of 70 (a threshold reported to ensure reliable backbone prediction) [71] and a maximum mean PAE of 5 to exclude artificial pockets caused by incorrect inter-domain orientations [15]. Finally, only pockets with at least 50% of their residues belonging to any given InterPro domain were kept, to ensure that they were located in biologically relevant regions rather than unannotated or disordered ones. Potential targets were then aligned in two ways. First, a ‘global’ alignment, using both full-sequence proteins. In cases where the target originated from the PDB subset, the same PDB structure was used, employing the chain(s) closest to the ligand or ligands, if more than one copy of the ligand was present in the structure. In cases where the closest chain did not correspond to a protein (e.g., nucleic acids), the alignment was not carried out. On the other hand, for targets originating from either ChEMBL or BindingDB, the UniProt accession was retrieved and the corresponding AlphaFold structure was subsequently obtained from AlphaFoldDB.

After the ‘global’ alignment, ‘local’ alignment runs were performed using the predicted binding sites. For each binding site, a structure file was generated by extracting from the original *T. cruzi* AlphaFold structure all residues with at least one atom within 12 Å of the pocket centroid. For the target proteins, if they originated from PDB, the environment of the ligand was extracted from the structure, while for the rest, binding pockets were predicted using the same methods as in the construction of the database, and filtered using the same criteria as above. In both cases, a pocket file was generated by keeping all residues with an atom within 12 Å of the ligand (PDB) or pocket centroid (rest). The resulting files were then aligned with each *T. cruzi* predicted pocket file. This meant that for each protein we obtained one global and several (one per pocket) local alignment scores.

## 4. Conclusions

In conclusion, it is important to stress that the output of the pipeline are predictions that will require experimental validation. Its aim is not to definitively assign a molecular target or targets to a given queried compound, but to render a shortlist of targets that help guide experimental work, allowing lab researchers to focus available resources on the most promising candidates. Beyond *T. cruzi*, this pipeline could be easily adapted to other organisms by substituting the AlphaFold model database for that of, for example, *Leishmania* spp., without having to change the underlying methodology or the ligand database. This modularity makes it a potentially broadly applicable tool for target identification across kinetoplastids and other pathogens causing NTDs where experimental target deconvolution remains resource-prohibitive. The scripts used in this pipeline are available on https://github.com/isglobal-chagas/chagas-target-discovery.

## Figures and Tables

**Table 1:** Protein–ligand database description.

Database Origin	Number of Unique Compounds ^1^	UniProt Target Proteins ^1^	PDB Target Proteins
ChEMBL	515,901	5521	NA
BindingDB	374,408	3642	NA
PDB	33,566	19,752	65,674 (6820 “orphan”) ^2^
TOTAL	877,824	24,137	72,494

^1^ The individual numbers do not add up to their totals because some compounds have binding data in more than one database. ^2^ Total number of PDB entries. Any given protein (identified with a UniProt ID) can have multiple PDB entries. Some PDB entries were not possible to relate to a UniProt entry (“orphan” entries). PDB = Protein Data Bank.

**Table 2:** Compounds used in the pipeline and their previously described main targets.

Compound	Main Target	References
Benznidazole	Nitroreductase	[37]
Nifurtimox	Nitroreductase	[37]
GNF6702	Proteasome	[38]
17-DMAG	Heat shock protein 90	[39]
Acoziborole	CPSF3	[40]
AN2-502998	CPSF3	[41]
IID432	Topoisomerase II	[42]
AB1	CLK1	[43]
Hippeastrine	NA	[44]
Miltefosine	NA	[45]

NA = not applicable.

**Table 3:** Summarized pipeline results.

Compound	Database Hit	Hit Similarity	Rank	Hit Protein ID	Hit Protein Description	*T. cruzi* Protein ID	*T. cruzi* Protein Description	Global Score	Local Score
Nifurtimox	PDB U6Z	0.798	6	7NB9	*Escherichia coli* NfsA with nitrofurantoin	Q4DCW9	Nitroreductase, NADH dehydrogenase	0.656	0.571
	PDB O3D	0.797	7	8PL9	Thioredoxin glutathione reductase of *Schistosoma mansoni*	Q4CT43	Trypanothione reductase	0.759	0.289
Benznidazole	CHEMBL1521315	0.861	3	P11511	*Homo sapiens*|aromatase	Q4D0S6	Cytochrome P450	0.785	0.526
	CHEMBL147183	0.838	8	P49430	*Rattus norvegicus*|thromboxane-A synthase	Q4D0S6	Cytochrome P450	0.784	0.460
	CHEMBL418043	0.824	15	P24557	*H. sapiens*|thromboxane-A synthase	Q4D0S6	Cytochrome P450	0.783	0.512
	PDB WDU	0.817	20	4WDU	17beta-HSD5	Q4D831	Aldo/keto reductase	0.926	0.616
GNF6702	PDB N2E	0.993	1	6TCZ_K	*Leishmania tarentolae* proteasome 20S subunit	Q4CU77	Proteasome subunit beta type-2	0.992	0.802
	PDB N2E	0.993	1	6TCZ_L	*L. tarentolae* proteasome 20S subunit	Q4D6T6	Proteasome subunit beta type-5	0.983	0.237
17-DMAG	PDB KOS	1.000	1	1OSF	Human Hsp90	Q4DW89	Heat shock protein 90	0.915	0.744
	PDB KOS	1.000	1	P41148	*Canis lupus familiaris*|endoplasmin	Q4DBM7	Heat shock protein 85	0.790	0.893
	PDB KOS	1.000	1	O15054	*H. sapiens*|lysine-specific demethylase 6B	Q4CXU5	JmjC domain, hydroxylase	0.178	0.840
	PDB KOS	1.000	1	P29375	*H. sapiens*|lysine-specific demethylase 5A	Q4CXU5	JmjC domain, hydroxylase	0.172	0.555
	CHEMBL3104859	1.000	2	P08238	*H. sapiens*|heat shock protein hsp 90-beta	Q4DBM7	Heat shock protein 85	0.900	0.918
	CHEMBL3104859	1.000	2	Q12931	*H. sapiens*|heat shock protein 75 kDa, mitochondrial	Q4E2Q2	Heat shock protein	0.810	0.813
	CHEMBL3104859	1.000	2	P14625	*H. sapiens*|endoplasmin	Q4DBM7	Heat shock protein 85	0.792	0.910
	PDB D1S	0.987	3	3Q5J	*Leishmania major* HSP90	Q4DW89	Heat shock protein 90	0.919	0.739
	PDB 8TO	0.986	4	4ASB	Yeast N-terminal Hsp90	Q4DW89	Heat shock protein 90	0.920	0.743
	PUBCHEM 6505803	0.982	6	Q58FG1	*H. sapiens*|putative heat shock protein hsp 90-alpha A4	Q4DBM7	Heat shock protein 85	0.705	0.284
	PDB GDM	0.954	7	4XDM	*Dictyostelium discoideum* N-terminal domain of Hsp90	Q4DW89	Heat shock protein 90	0.918	0.760
	PDB BC2	0.949	8	3PEJ_A	*Plasmodium falciparum* N-terminal domain of HSP90	Q4DBM7	Heat shock protein 85	0.758	0.718
	PDB BC2	0.949	8	3PEJ_B	*P. falciparum* N-terminal domain of HSP90	Q4DW89	Heat shock protein 90	0.756	0.706
Acoziborole	PDB XYX	0.841	11	8T1Q	Human CPSF73 catalytic segment	Q4DR37	Cleavage and polyadenylation specificity factor	0.840	0.264
AN2-502998	NA ^1^								
IID432	NA ^1^								
AB1	PDB 5XH	1.000	1	6Q2A	*Trypanosoma brucei* CLK1 kinase domain	Q4E3Z0	Kinetoplastid kinetochore protein 19	0.986	0.838
Hippeastrine	PDB 3KD	0.914	2	4U4U	Yeast 80S ribosome	Interacting with nucleic acids			
	PDB HN8	0.898	3	5ON6	80S ribosome	Interacting with nucleic acids			
	PUBCHEM 65305	0.871	4	Q16539	*H. sapiens*|mitogen-activated protein kinase 14	Q4D3A0	Mitogen-activated protein kinase 2	0.886	0.777
	PDB A1L8F	0.870	5	9M0P	Human 80S ribosome	Interacting with nucleic acids			
	PDB MQ6	0.820	19	7UCJ	Mammalian 80S translation initiation complex	Interacting with nucleic acids			
Miltefosine	PDB C6W	0.900	1	2YP6	*Streptococcus pneumoniae* thioredoxin	Q4CX87	Peroxiredoxin	0.834	0.172
	CHEMBL372764	0.887	2	P15374	*H. sapiens*|ubiquitin carboxyl-terminal hydrolase isozyme L3	Q4D1R0	Cysteine peptidase, Clan CA, family C12, ubiquitin carboxyl-terminal hydrolase	0.802	0.299
	CHEMBL462609	0.816	8	O35678	*Mus musculus*|monoglyceride lipase	Q4CSM0	Monoglyceride lipase	0.891	0.809
	CHEMBL462609	0.816	8	O08914	*M. musculus*|fatty-acid amide hydrolase 1	Q4D5K8	Amidase	0.717	0.582
	PDB MAY	0.813	13	1MT5	Rat fatty acid amide hydrolase	Q4D5K8	Amidase	0.765	0.542
	CHEMBL113262	0.813	14	Q99685	*H. sapiens*|monoglyceride lipase	Q4CSM0	Monoglyceride lipase	0.864	0.588
	CHEMBL113262	0.813	14	O00519	*H. sapiens*|fatty-acid amide hydrolase 1	Q4D5K8	Amidase	0.718	0.619
	PDB 4E0	0.811	16	7P0Y	*Mycobacterium tuberculosis* mtbMGL	Q4CSM0	Monoglyceride lipase	0.936	0.554

^1^ The previously described targets for AN2-502998 and IID432 did not feature among the top 20 matches.

## Data Availability

The scripts used in this pipeline are available on https://github.com/isglobal-chagas/chagas-target-discovery. Raw pipeline results are provided as Supplementary File S1.

## Supplementary Materials

The following supporting information can be downloaded at: https://www.mdpi.com/article/10.3390/ph19071046/s1, Supplementary File S1: Raw pipeline results provided as a CSV file; Supplementary Figure S1: Flowchart illustrating the filtering process used to select *T. cruzi* proteins for inclusion in the pipeline; Supplementary Figure S2: Distribution of the 15 most frequently represented protein families in the ChEMBL dataset. Protein family assignments were derived from the level 2 target classification provided by ChEMBL for each protein, where available.

## Abbreviations

The following abbreviations are used in this manuscript:

BNZ	Benznidazole
CD	Chagas disease
CPSF	Cleavage and polyadenylation specificity factor
CRISPR	Clustered regularly interspaced short palindromic repeats
EC_50_	Half-maximal effective concentration
ErG	Extended-Reduced Graph
Hsp	Heat shock protein
IC_50_	Half-maximal inhibitory concentration
$K_{d}$	Dissociation constant
$K_{i}$	Inhibition constant
NFX	Nifurtimox
NTD	Neglected tropical disease
NTR	Nitroreductase
PAE	Predicted aligned error
PDB	Protein Data Bank
pLDDT	Predicted local distance difference test
QSAR	Quantitative structure–activity relationship
RNAi	RNA interference
SMILES	Simplified Molecular Input Line Entry System
