# Supplementary material for: Integrating Molecular Similarity and AlphaFold-Based Structural Alignment for Target Discovery in Trypanosoma cruzi
Source: Pharmaceuticals (Basel). 2026 Jul 7;19(7):1046. doi: 10.3390/ph19071046 (PMC13414571; doi:10.3390/ph19071046)
Supplement: Supplementary file 1 [file pharmaceuticals-19-01046-s001.zip › pharmaceuticals-4355193-supplementary.pdf]

pharmaceuticals

Article

Integrating Molecular Similarity and AlphaFold-Based Structural Alignment for Target Discovery in *Trypanosoma cruzi*

Albert Ros-Lucas [1,2,3,*], Nieves Martínez-Peinado [1,3,4], Juan Carlos Gabaldón-Figueira [1,3,5], Maria Morillo-Osorio [1], Cristina Ballart [1,3,4], Montserrat Gállego [1,3,4], María-Jesús Pinazo [3,6], Joaquim Gascón [1,3], Ana Requena-Méndez [1,3,7] and Julio Alonso-Padilla [1,3,8,*]

1 ISGlobal, 08036 Barcelona, Spain
2 Campus Mar, Universitat Pompeu Fabra (UPF), 08003 Barcelona, Spain
3 CIBERINFEC, ISCIII–CIBER de Enfermedades Infecciosas, Instituto de Salud Carlos III, 28029 Madrid, Spain
4 Secció de Parasitologia, Departament de Biologia, Sanitat i Medi Ambient, Facultat de Farmàcia i Ciències de l'Alimentació, Universitat de Barcelona, 08028 Barcelona, Spain
5 Department of Clinical Microbiology, Hospital de la Santa Creu i Sant Pau, 08025 Barcelona, Spain
6 Drugs for Neglected Diseases Initiative (DNDi), Rio de Janeiro 20010-020, Brazil
7 Department of Medicine Solna, Karolinska Institute, 171 77 Stockholm, Sweden
8 INTERTRYP, University of Montpellier, CIRAD, IRD, 34398 Montpellier, France
* Correspondence: albert.ros@isglobal.org (A.R.-L.), julio.a.padilla@isglobal.org (J.A.-P.)

Supplementary File S1: Raw pipeline results provided as a CSV file. The columns are defined as follows:

- `query_mol`: Identifier of the query molecule.
- `chem_id`: Chemical database identifier of the matched compound.
- `smiles`: SMILES representation of the matched compound.
- `pattern_cosine`: Cosine similarity based on the Pattern fingerprint.
- `erg_cosine`: Cosine similarity based on the ERG fingerprint.
- `pattern_scaffold_cosine`: Cosine similarity based on the scaffold Pattern fingerprint.
- `similarity_score`: Weighted molecular similarity score used for ranking.
- `database`: Source database of the matched compound.
- `hit_protein_id`: Identifier of the matched (hit) protein.
- `hit_protein_name`: Name of the matched protein.
- `hit_protein_chain`: Chain identifier of the matched protein structure.
- `hit_protein_pocket_id`: Identifier of the predicted binding pocket in the matched protein.
- `hit_protein_pocket_score`: P2Rank score of the predicted binding pocket.
- `hit_protein_affinity`: Reported binding affinity associated with the matched protein–ligand complex.
- `target_protein_id`: Identifier of the *T. cruzi* protein.
- `target_protein_name`: Name of the *T. cruzi* protein.
- `target_protein_pocket_id`: Identifier of the predicted binding pocket in the *T. cruzi* protein.
- `target_protein_pocket_score`: P2Rank score of the predicted binding pocket.
- `global_rmsd`: RMSD of the global structural alignment between the two proteins.
- `global_seq_id`: Sequence identity over the global protein alignment.
- `global_score`: TM-score of the global structural alignment.
- `local_rmsd`: RMSD of the local structural alignment.

- `local_seq_id`: Sequence identity of the local structuralalignment.
- `local_score`: TM-score of the local structural alignment.

Supplementary Figure S1: Flowchart illustrating the filtering process used to select *T. cruzi* proteins for inclusion in the pipeline.

Supplementary Figure S2: Distribution of the 15 most frequently represented protein families in the ChEMBL dataset. Protein family assignments were derived from the level 2 target classification provided by ChEMBL for each protein, where available.

https://doi.org/10.3390/ph19071046
