# Supplementary figures and images for: Integrating Molecular Similarity and AlphaFold-Based Structural Alignment for Target Discovery in Trypanosoma cruzi
Source: Pharmaceuticals (Basel). 2026 Jul 7;19(7):1046. doi: 10.3390/ph19071046 (PMC13414571; doi:10.3390/ph19071046)

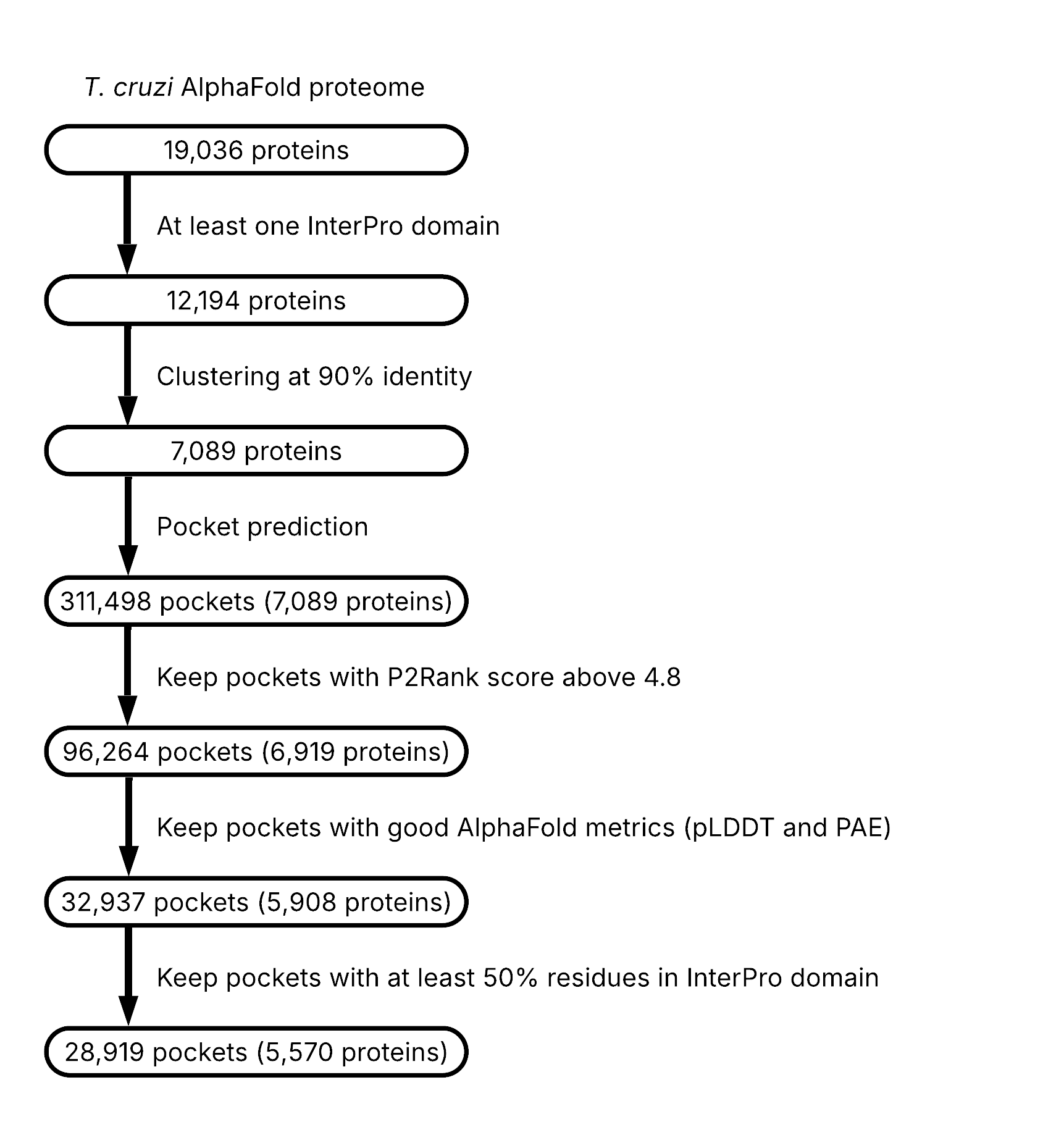

Supplement: Supplementary file 1 [file pharmaceuticals-19-01046-s001.zip › Supplementary_Figure_S1.png]

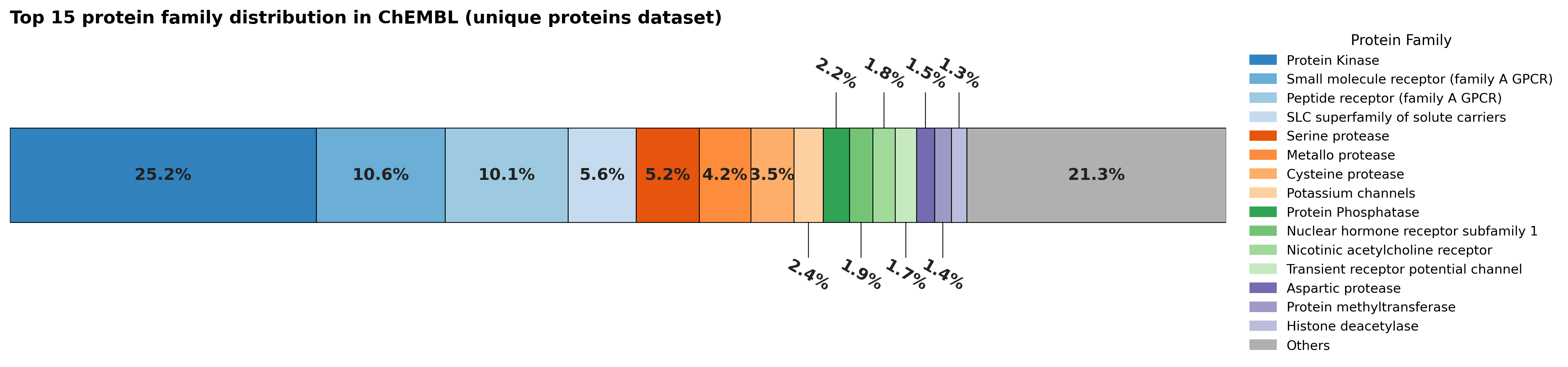

Supplement: Supplementary file 1 [file pharmaceuticals-19-01046-s001.zip › Supplementary_Figure_S2.png]
